# Control Methods for Transradial Prostheses Based on Remnant Muscle Activity and Its Relationship with Proprioceptive Feedback

**DOI:** 10.3390/s20174883

**Published:** 2020-08-28

**Authors:** Stefan Grushko, Tomáš Spurný, Martin Černý

**Affiliations:** Department of Robotics, VSB-Technical University of Ostrava, 70800 Ostrava, Czech Republic; tomas.spurny.st@vsb.cz (T.S.); martin.cerny@vsb.cz (M.Č.)

**Keywords:** prosthetic control, prosthetics, biosensing, proprioception, proprioceptive feedback

## Abstract

The loss of a hand can significantly affect one’s work and social life. For many patients, an artificial limb can improve their mobility and ability to manage everyday activities, as well as provide the means to remain independent. This paper provides an extensive review of available biosensing methods to implement the control system for transradial prostheses based on the measured activity in remnant muscles. Covered techniques include electromyography, magnetomyography, electrical impedance tomography, capacitance sensing, near-infrared spectroscopy, sonomyography, optical myography, force myography, phonomyography, myokinetic control, and modern approaches to cineplasty. The paper also covers combinations of these approaches, which, in many cases, achieve better accuracy while mitigating the weaknesses of individual methods. The work is focused on the practical applicability of the approaches, and analyses present challenges associated with each technique along with their relationship with proprioceptive feedback, which is an important factor for intuitive control over the prosthetic device, especially for high dexterity prosthetic hands.

## 1. Introduction

Even though prosthetics as a field of medicine originated many centuries ago, it is still an area that is developing and largely dependent on the research of new technologies. In the field of prosthetics, there has been significant progress in recent decades due to the development of biomechanics, neurology, new materials, and approaches. However, even the most modern innovations and discoveries in this area cannot provide an ideal replacement for lost limb functions.

Multiple control topologies have been developed over the past seventy years with electromyography (EMG) being the most commonly used technology these days. However, there are other less-known and yet unproven techniques available, which may potentially represent a number of advantages such as simultaneous proportional control over multiple degrees of freedom (DOFs) and the possibility of enabling intuitive proprioceptive feedback.

This paper represents an extensive review covering existing biosensing approaches for the implementation of control for transradial prostheses, focusing on their applicability and discusses possible implementations of hybrid techniques. The goal is to provide indications for the development of future prosthetic technologies and to facilitate future research in this area. The work is focused on the control methods based on the monitoring of physiological changes in residual muscles during their contraction. It also analyses their relationship with an important factor for intuitive control of prosthetic devices: proprioceptive feedback. The expected value additions from this work are to give information about currently existing standard and promising techniques, by analyzing some of the available results and pointing out key aspects.

The paper is organized as follows: Section 2 introduces necessary background information to the topics of amputation, proprioception, and prostheses control. Section 3 presents available control approaches proven for prosthesis control, along with a brief analysis of their advantages, disadvantages, and possible directions for future research. Section 4 continues presenting the control approaches by describing promising techniques, which have not been tested yet for controlling prosthetic devices. Section 5 will briefly present advances in enabling intuitive proprioceptive feedback for the users of the prosthetic devices. Lastly, the summarization of the provided overview is discussed, and conclusions of the paper are provided.

## 2. Background

This section will present a brief background of the related topics of amputation, proprioception, and upper limb prosthetic control.

### 2.1. Amputation

**Figure 1:**
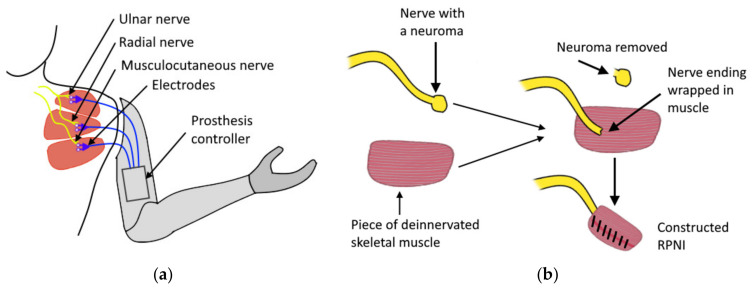
Surgical procedures for improving the control over a prosthesis: (**a**) TMR—reinnervated muscles might be used for prosthesis control after proximal amputation [10]; (**b**) illustration of RPNI construction [5].

Depending on the amputation level, upper extremity amputation can be classified into partial hand amputation, wrist disarticulation, transradial, elbow disarticulation, transhumeral, shoulder disarticulation, and forequarter amputations. The choice of applicable control topology for a prosthesis depends on the level of amputation. This paper focuses on the section of transradial prostheses. Transradial (also known as “below elbow”) amputation occurs through the radius and ulna of the lower arm. After an amputation of this level, finger flexors and wrist flexors (also corresponding extensors) located in the forearm are often available and represent possible sites to be used to get control signals for a hand prosthesis. In cases when the amputation is very proximal, the muscles of the amputated region are lost, complicating the application of biosensing techniques based on the monitoring of activity in the residual muscles. Separate surgical operations such as Targeted Muscle Reinnervation (TMR, Figure 1a) [1,2] and Regenerative Peripheral Nerve Interface [3,4,5] (RPNI, Figure 1b) may be additionally performed to redirect and engraft the remaining nerves of the amputated limb to a new location of healthy muscles (or muscle grafts in case of RPNI), sometimes making it possible to measure the activity in these muscles. Both approaches have also proved to be an efficient treatment [6,7,8] for painful neuromas that often appear in patients who have undergone amputation. Although usually applied following proximal amputations, the TMR technique is also used for patients with transradial amputations [9]. However, the topic of regenerative interfaces is broad, and its extensive presentation is beyond the scope of this article. 

An alternative to surgery would be to measure the activity in the muscles other than the ones used to control the limb initially, and, if the signals are strong enough, use them to control the prosthesis. However, both in case of surgery and while using the other muscles to get the control signals, it is not yet possible to establish a fully intuitive control over the prosthesis and the patient has to adapt to new associations. The adaptation process takes time, and the patient must regularly perform rehabilitation exercises to prevent muscle atrophy [11].

### 2.2. Proprioception

Important aspects affecting the ease of use of prostheses are the intuitiveness of operation and the feedback system for the coordination of movements [12,13]. The absence of sensory feedback from the limb affects and complicates the daily use of the prosthesis. However, tactile information alone is not enough for intuitive control. Another critical aspect for the intuitive and agile operation of the prosthesis is the ability of the user to have proprioceptive feedback on all movements performed.

Proprioception, kinaesthesia, and neuromuscular control are terms that are often used interchangeably in literature; however, these terms have distinctive definitions. Proprioception is the awareness of joint position, whereas kinaesthesia is the awareness of joint movement [14]. Neuromuscular control is defined as the unconscious trained response of a muscle involved in the control of dynamic joint stability. Proprioceptive sensory signals are critical for the control of movement [15,16] and represent an entirely distinct sensory modality from touch [17] (contact and pressure sensations). The senses contributing to proprioception are hard to evaluate, measure, or compare [18,19,20,21,22] due to their partially non-conscious nature. A series of tests conducted by Han et al. [23,24] ascertained that the proprioception ability of human joints is site-specific, for example right handed individuals have demonstrated consistently better proprioceptive abilities for the joints on the left side of the body (when compared with the abilities on the right side). 

Proprioceptive sensory signals are generated by various mechanoreceptors (proprioceptors) located in muscles, joint capsules, and ligaments, which together provide sensory input to the central nervous system [14]. Golgi tendon organs are proprioceptors that lie at the interface between muscles and tendons and activate at certain muscle forces indicating the resistance that muscle is experiencing. The primary muscle receptors that contribute to the sense of position and motion are muscle spindles, which are embedded in skeletal muscle fibres. The primary endings of muscle spindles are sensitive to the velocity and extent of muscle stretch. The secondary endings provide information about the actual muscle length. Proprioceptors responsible for detecting the motion thresholds of the joints are Ruffini endings (skin tension receptors) and Pacinian corpuscles (embedded in the joint capsules). Signals from the muscle spindles, skin, and joint receptors are combined to provide comprehensive information about the joint movement and position to the central nervous system [25].

Dynamic relationships within agonist–antagonist muscle pairs in a limb are also fundamental for creating a natural sensation of joint movement [26] since this connection engages the related proprioceptors. During a typical amputation procedure, muscle tissues in the residual limb are placed isometrically severing the dynamic connection between agonist–antagonist muscle pairs, which limits the ability of the muscles to provide meaningful proprioceptive feedback [27]. 

Multiple studies [17,28,29,30] have been undertaken to cover the role of proprioceptive feedback in the control of upper-limb prostheses. They conclude that proprioceptive sensations could significantly improve the ability to accurately perform tasks with a prosthesis with or without visual feedback. However, none of the currently available commercial prostheses is equipped with a sufficient feedback system to at least partially compensate the user with all senses included in the concept of proprioception. The user has to coordinate the movements of the prosthesis relying entirely on exteroception [13]—the sensations that result from stimuli originating outside the user’s body, which in the case of hand prostheses is usually vision. However, the somatosensory system provides not only proprioception but also information about touch, pain, temperature, and vibrations. All types of sensations are important for the sense of agency, prosthetic embodiment, and control in general. Generally, simultaneous integration of multi-modal senses into prostheses currently is unachievable due to its complexity. The topic of sensory feedback in prosthetic devices was comprehensively covered in [31,32].

### 2.3. Prosthesis Control

Depending on the interaction between the user and the device, the hand prostheses currently available in the market can be divided into two types, passive and active prostheses. The passive prostheses are designed only for aesthetic purposes and can be used for a very limited range of tasks. The active prosthetic devices can be further divided into body-powered and externally powered. Since body-powered prostheses offer only minimal control over the movement, the electronic prostheses are a common option for patients willing to use a prosthesis [12]. A poorly designed or improperly configured prosthesis may not only be limited by what it can do, but it can also effectively endanger the life of the patient. This is why the control systems used in the externally powered arm prostheses are based on proven technologies that are desirably simple and reliable at the tasks they are designed to perform. Most of the currently known methods of prosthesis control rely on getting measurements on the physiological properties of the remnant muscles (or reinnervated proximal muscles after TMR) in the stump and converting these measurements into appropriate control signals for the prosthesis. In this case, the muscles act as biologic amplifiers for the neural commands, which are then picked by the sensor. The approaches utilizing the bio-signals from the peripheral or central nervous system and enabling bidirectional communication link (brain–machine interface) are still in their early days of development and currently possess multiple disadvantages [33,34]. These methods are not the subject of this review. 

Control strategies for prosthetic devices define how the muscle activation signals obtained from sensing techniques are utilized to control the prosthesis and drastically influence the ease of use of the prosthetic device [35]. Typically, upper limb prostheses utilize proportional, pattern-based control strategies and a variety of their combinations. The proportional control strategy may enable position, velocity, or force control over the chosen DOFs of the prosthesis, making the controlled motion parameter proportional to the degree of muscle contraction. Although direct proportional control of prosthetic fingers can be considered more natural than the grip selection approach, it could be more difficult for the user to appropriately adjust the position of the fingers for the task considering that usually, only a limited number of control sites are available [13]. Additionally, proportional control of the prosthesis requires feedback (closed-loop control) to enable the user to use it without constant visual inspection. The pattern-based control strategies enable the user to switch between a predefined set of gestures. The combination of proportional and pattern-based control strategies allows the user to control the level of execution of predefined grasp motion proportionally. According to the assessment of user-needs [13], it is prefered for the prosthesis to enable the user with control of force, speed (ideally both), as well as simultaneous control over multiple prosthetic joints [13]. Control strategies for upper-limb prosthesis are covered in detail by work of Roche et al. [35].

Most of the biosensing control techniques that are discussed further in the paper can enable sensing at multiple target sites (multiple muscles). For proportional control strategy, each target site may be responsible for actuating its own DOF of the prosthesis. In the case of pattern-based control, the synergy of the signals from different sites may be used for identification of a specific muscle activation pattern. For instance, a series of prosthetic hands i-Limb [36] from Össur utilizes two-site (wrist flexors, extensors) surface electromyography measurements as an initial input for pattern-based control. It further utilizes muscle activation patterns (such as double, triple muscle contractions, and simultaneous contractions of opposing muscles) to increase the number of grips supported. The result of each pattern activation may be configured by the user (switch to a specific grip or switch to control of a different prosthesis DOF).

The biosensing-based control approaches can be categorized into invasive and non-invasive approaches. Invasive approaches imply surgical procedure to implant electrodes or otherwise change the physiological characteristics of the stump. Non-invasive approaches, on the other hand, utilize technologies enabling indirect measurements of physiological parameters without an actual contact of the device with the monitored muscle or nerve ending. Considering that the alternative is a surgery, the non-invasive methods are preferred by most users [12] even though those methods are usually less accurate than invasive ones [37]. Another categorization can be based on the origin of the control signal. The majority of currently used methods (direct and indirect) measure changes of physiological parameters of muscles during activity to obtain control signals for the prosthesis [38]. In the meantime, the actively developing field of the brain–machine interface and connection to peripheral nervous system represents a different set of approaches [39,40]. This review focuses on the prevalent group of biosensing methods based on the measurement of the activity of residual muscles. Techniques covered in this paper also include methods for hand gesture recognition, since they are capable of recognizing the intended gestures (by monitoring the changes in residual stump) of amputees and thus providing control means for prosthesis even though initially they were intended for use by able-bodied users. The paper does not cover the data processing, since it depends on the control strategy and the chosen biosensing technique itself. Feature extraction, data processing, and signal classification techniques are covered by the work of Cloutier and Yang [41] and will not be covered in this paper. 

The control techniques briefly presented in this review will be separated into two sections depending on their testing in the prosthetic applications and the physical basis of their implementation. Section 3 will present non-invasive techniques (with possible invasive alternatives) for monitoring muscle activity which were proven viable for transradial prosthesis control. Section 4 includes gesture prediction techniques and concepts of control topologies, both of which have not been tested for prosthesis control yet. The emphasis is put on describing methods for transradial prostheses; however, since at lower levels of amputation more biological structures are available, the presented methods, with some degree of modification, may be suitable for other (both proximal and distal) amputations.

## 3. Approaches Proven for Prosthetic Control

This part will cover biosensing techniques proven to be applicable for establishing control over an externally powered prosthetic device. Each method has its limitations, and their applicability depends on physiological parameters of the patient’s stump. None of the methods presented in this section provides proprioceptive feedback to the user.

### 3.1. Electromyography

**Figure 2:**
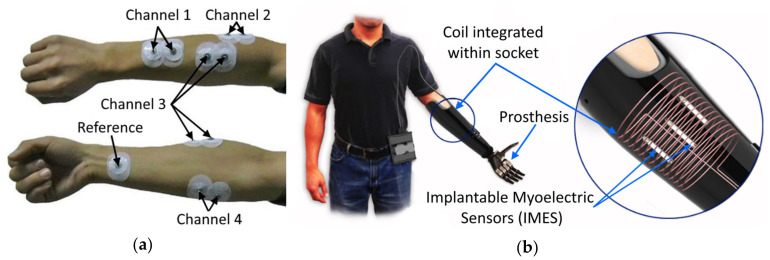
Electromyography: (**a**) illustration of typical mid-arm EMG measurement setup for four independent sEMG channels [46]. Adapted from [46], licensed under CC BY 4.0; (**b**) intramuscular electromyography with muscle-implanted wireless electrode modules (Implantable Myoelectric Sensors). This figure was adapted from [45] with permission from Elsevier.

Although multiple control methods have been developed in recent years, standard hand prostheses commonly use electromyography (EMG)–an approach based on measuring electric potentials generated by muscles during contractions. The EMG signal’s amplitude lies in between 1–10 mV with the frequency range up to 500 Hz and most signal energy in between 50–150 Hz [42]—in case the electric muscle potentials are measured on the surface of the skin using dry or adhesive electrodes, and this method is referred to as surface electromyography (sEMG). An example of surface electrode placement for monitoring of wrist flexors and extensors is shown in Figure 2a. Intramuscular electromyography (iEMG) implies placing the sensing electrodes over the muscle under the skin [43] or implanting electrodes directly [44,45] into the targeted muscle. In the latter case, the electrodes may be in the form of an independent miniature implantable module capable of wirelessly transmitting the measured signal (see Figure 2b). Both sEMG and iEMG electrodes are usually placed on (or inside) large muscle groups in order to achieve a better signal-to-noise ratio. EMG electrodes are typically placed between the motor unit and the tendinous insertion of the muscle [42]. The signal collected by these electrodes is then filtered, amplified, and can then be used as a control signal for the prosthesis. 

Depending on the number of measurement sites, possible EMG signal acquisition configurations include [42]:

- Monopolar configuration acquiring a signal using a single electrode with respect to a reference (ground) electrode usually placed on an electrically neutral tissue (in the vicinity of a bone).
- Bipolar configuration—the signal is acquired using two active electrodes (placed 1–2 cm from each other) with the help of a reference electrode. The signals from the two measuring sites are collected with respect to a common ground provided by the reference electrode and then amplified. This helps in the mitigation of ambient electromagnetic noise.
- Multipolar configurations—similar to the bipolar configuration but uses more than two active electrodes. This configuration further reduces crosstalk and noise.

Comprehensive practical recommendations for EMG signal acquisition and processing are provided by the work of De Luca [42].

For EMG, the overall accuracy of gesture prediction depends on the number of sensors, which was shown in the works of Muceli et al. [47] and Amma et al. [48]. The latter introduced a compensation approach for a shift in the position of the electrodes by identifying the location of ulna, where no muscles are present and thus very low EMG activity is registered (by an array of 192 high-density electrodes uniformly placed along forearm surface), allowing an accuracy of 90% at the task of recognizing a set of 27 gestures. The intramuscular approach in a series of experiments [37] has proven to be more accurate. However, both the approaches possess inherent drawbacks, such as signal degradation due to muscle fatigue [49], relatively poor spatial resolution caused by adjacent muscle crosstalk (especially for large electrodes [42,50]), and susceptibility to electromagnetic noise. The last two disadvantages are mitigated for implantable electrode modules [51]. In the sEMG measurement approach, the position shifts of the sensors between measurement sessions may have an adverse effect on the accuracy (this factor is also mitigated for implantable electrodes). Additionally, the sEMG method is only able to record signals from superficial muscles, whose function frequently does not relate to the intended prosthetic function. Although the approach with implantable electrodes is more accurate, the invasiveness has impeded the user acceptance of the method [52]. An apparent approach of improving the control performance by adding more EMG sensor nodes is not always available due to insufficient surface area for placing the electrodes. Even though high-density sEMG recordings provide superior results, currently the required equipment is bulky and not suitable for compact design typically required for prosthetic devices. 

### 3.2. Electrical Impedance Tomography

**Figure 3:**
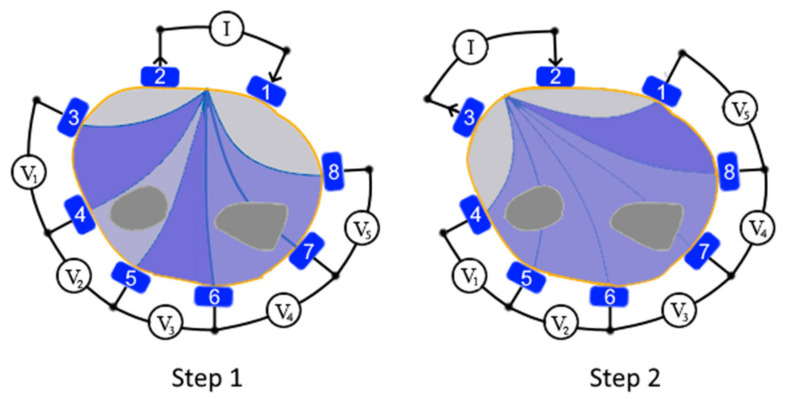
Illustration of two excitation steps during measurement of cross-sectional impedances between pairs of electrodes (four-pole measurement schemes with eight electrodes). Measurement is repeated for all electrode pairs. A higher voltage difference is shown in a brighter blue colour. Objects with a high impedance within the cross-section are shown in grey.

Electrical Impedance Tomography (EIT) [53,54] is a non-invasive, radiation-free bio-impedance mapping technique. It utilizes an array of surface electrodes wrapped around a part of the user’s body to measure the electrical impedance of the tissues in the cross-section plane covered by electrodes. The measurement is performed by exciting a sine wave current (with frequencies in the range of 10 kHz up to 1 MHz [55]) using a pair of surface electrodes and recording the voltages on the surface of the monitored cross-section of an object with the remaining electrodes. The changes of amplitude and phase of these recorded voltages represent the changes of distribution of internal conductivity within the cross-section. All electrodes for EIT are required to have a conductive contact with the skin. Multiple EIT measurement strategies are available with the two most prevalent being two-terminal and four-terminal schemes [56]. The two-terminal scheme assumes the use of a pair of electrodes to perform the impedance measurements. One of the electrodes acts as the emitter, and the other operates as the receiver (the process is repeated for each pair of electrodes). This approach is frequently used due to its simplicity, although it is more susceptible to variations in skin contact, thereby requiring the use of larger electrodes. In the four-terminal scheme, an adjacent pair of electrodes is excited with an AC signal while measuring the voltage between another pair of the available electrodes (principle illustrated in Figure 3). This process is repeated for all the electrode pairs. This differential measurement approach makes four-pole scheme less sensitive to contact conditions on the skin enabling the use of a larger number of small electrodes [57] to improve the quality of the measurements. 

Due to the non-invasive nature of the method, it has been widely used in clinical applications such as sensing changes in lung ventilation, brain function, and blood flow. For hand gesture recognition, the electrodes are typically wrapped around the forearm near to the wrist; however, any position along forearm is acceptable (as demonstrated by Wu et al. [58] for control of a hand prosthesis) as long as the changes in impedance are large enough to be registered by the system and distinguished as separate gestures. Zhang et al. proposed [59] a wearable wrist-worn EIT device, which adopted a two-terminal measurement scheme with eight electrodes to recognize a set of eight hand gestures in real-time while achieving a high accuracy (96% when worn on the wrist and 93% for proximal forearm placement). The researchers further improved the system [60] by enabling different measurement configurations including modes with 8, 16, and 32 electrodes and switching between two-terminal and four-terminal measurement schemes. The configurations were compared, and the highest overall accuracy of the reconstructed map was obtained by the configuration with 32 electrodes and a four-terminal measurement scheme. However, this configuration led to significantly slower measurement and processing times. Another consequence of increasing the number of electrodes is that each electrode must be smaller to accommodate the others, leading to a smaller contact area with the skin. This makes the electrodes more susceptible to variation in skin contact conditions. Furthermore, the method is susceptible to electromagnetic interference, which was partially solved by the researchers by switching to the excitation signal to a static frequency of 40 kHz. Researchers noted that the approach had a high susceptibility to the displacement of the electrodes during different measurements sessions, which suggest that a calibration procedure needs to be developed. In a long measurement series, calibration of the system would have to be readjusted to compensate for changes in electrode impedance, such as skin conductivity altered by sweating [61]. Future implementations of wearable devices may utilize measurement sequences with variable excitation signals of different frequencies (multi-frequency EIT [62]). Each excitation frequency may provide additional details about the tissue cross-section since different types of human tissue exhibit different values of bio-impedance. 

### 3.3. Near-Infrared Spectroscopy

**Figure 4:**
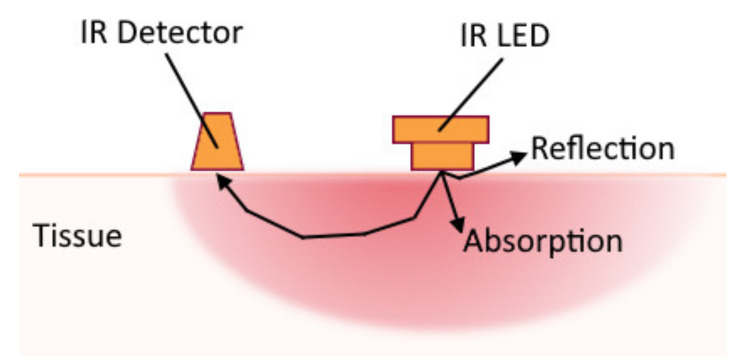
NIRS principle [65]. Infrared (IR) light is continuously emitted into muscle tissues by LED. The infrared light detector measures the amount of infrared light scattered by the tissue. The remaining portion of the light is reflected and absorbed by the surrounding tissues.

Human tissues are relatively transparent to light in the near-infrared range between 700 and 900 nm [63]. The main absorbers of this spectrum range are blood chromophores of oxygenated and deoxygenated haemoglobin (wavelengths 760 nm and 850 nm, respectively [64]). Near-infrared spectroscopy (NIRS) allows monitoring muscle perfusion and oxygenation during contractions. The commonly used approach to measure the NIRS signal is to combine a near-infrared LED with a photodetector (see Figure 4). 

In a standard measurement scheme, the LED emits near-infrared light into the tissue, while the photodetector measures the amount of light scattered in the nearby tissues. Muscle contraction changes the amount of blood in the tissue. This, in turn, changes the amount of near-infrared light that is scattered back to the skin surface detectable by the sensor. An example of NIRS-based gesture recognition was presented by Paleari et al. [64]. The experiment consisted of recognizing three gestures using single NIRS unit combined with single channel sEMG sensor (placed over wrist and finger extensor muscles) and comparing their results, which have indicated higher accuracy of NIRS approach (achieving 92% accuracy) for the specified task. The approach also allowed coarse estimation of the force applied to the movement.

It worth mentioning that the NIRS approach is closely related to Photoplethysmography (PPG), which is commonly used for heart rate detection in wearable devices (smartwatches). PPG differs from NIRS by the type of LED used and has also been proven as a feasible method for hand gesture recognition as it allowed for an average accuracy of 89% while predicting nine different gestures [66]. The wearable device was equipped with two PPG sensors and a single accelerometer for compensation of the signal artefacts caused by the hand motion.

It is possible to control the depth of the detectable NIRS signal by changing the distance between the light source and the detector. In a series of experiments it was shown that the region monitored under the skin is approximately half of the source-detector distance [67]. Thus, the chosen depth of the detection may limit the spatial resolution of the method.

One of the side-effects to be considered is heating of the tissue caused by the infrared LED. To minimize this effect and to prevent injuries, a trapezoidal-shaped activation signal can be used to create periodic pulses with the light source [68]. The reflected signal is susceptible to ambient light, so the detection area must be covered with opaque material [68] in such a way that ambient light is minimized. The application of the method requires a good contact of the sensors with the skin, since, without contact, the light may be reflected without penetrating the tissues and cause incorrect readings. NIRS, likewise surface EMG, is most effective for recording signals from superficial muscles limiting its applicability for prosthesis control. An apparent advantage of the method comparing to EMG is its immunity against electronic inference.

An early attempt of NIRS-based control for a prosthetic hand was demonstrated by Bianchi et al. [69]; however, the accuracy was not reported. Other research groups [65,68,70] attempted to combine NIRS with sEMG technique to achieve a more reliable control signal for control of a prosthetic device. Since NIRS can be used to measure muscle fatigue, its data can be potentially used to improve the accuracy of other biosensing techniques that are susceptible to signal degradation due to muscle fatigue (such as EMG).

### 3.4. Sonomyography

**Figure 5:**
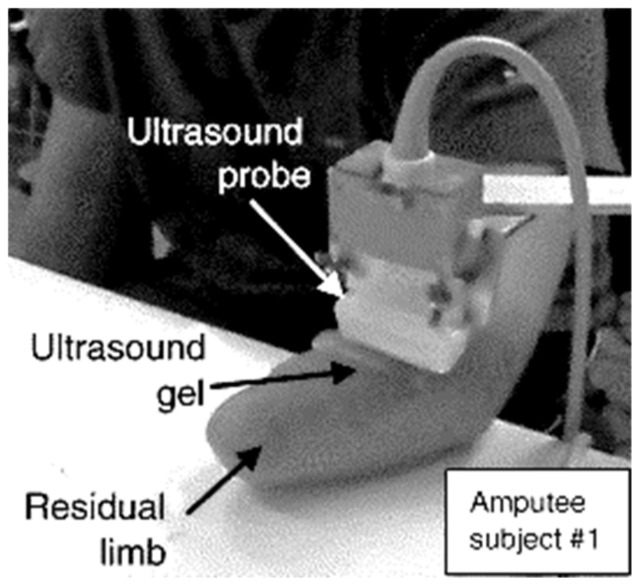
An example of a setup for collecting ultrasound images from the residual forearm of an amputee using a linear ultrasound probe. This figure was adapted from [71] with permission from Elsevier.

Sonomyography [71] (SMG) is a method of monitoring morphological changes in muscles using reflected ultrasound waves generated by piezoelectric transducers. The properties of the reflected signal are related to the acoustic impedance of the tissue. This makes it possible to create a map of the density distribution in the cross-section of the tissue using multiple transducers simultaneously. Ultrasound imaging provides a non-invasive interface allowing the monitoring of movements in both superficial and deep muscles (depending on the imaging depth) as well as tendons. One of the first studies utilizing ultrasound imaging for prosthesis control were works of Zheng et al. [71] and Shi et al. [72] in which the method was used for extracting muscle thickness for consecutive estimation of joint angle and torque. Multiple experiments have been carried out showing that, by utilizing this method, it is possible not only to accurately predict the degree of flexion of limb joints but also the force applied by the muscle [73]. Castellini et al. in their works showed the presence of a linear correlation between forces produced by the finger flexors (in able-bodied users), their positions and gradient features in 2D ultrasound images of the transverse section of the forearm [73,74,75]. Akhlaghi et al. applied ultrasound imaging to classify set of 15 hand gestures in real-time, demonstrating the feasibility of implementing ultrasound imaging as a robust human–machine interface achieving an overall accuracy of 92% [76]. It worth noting that the training phase of the gesture classifier involved collecting data on different orientations of the arm while holding it in the air, so the collected data represented more variability in deformation of the muscular geometry due to compression, which is often a factor that decreases the accuracy of the prediction. Dhawan et al. in their research [77] utilized sonomyographic control of prosthesis, allowing users to control multiple predefined hand motions proportionally. In the abovementioned implementations, the ultrasound probe was placed on the ventral side of the wrist orthogonal to the axis of the forearm. An example of a probe placement for an amputee is shown in Figure 5. In work of McIntosh et al. [78], four placement positions of the ultrasound probe along the ventral side of the forearm were analyzed, and the best performance was achieved for wrist and mid-arm placement. Still, the most efficient position of the probe is highly individual and depends on the amputation.

In most papers covering ultrasound-based control, researchers use linear ultrasound probes consisting of linear arrays of ultrasound transducers (typically working in the frequency range of 2–13 MHz with up to 512 piezoelectric elements) to capture the 2D images of the internal body structures. This type of measurement is called B-mode scanning, where the brightness of each pixel represents the tissue density in that point of the body. This approach offers high spatial resolution (depending on the number of ultrasound transducers within the probe); however, probes with multiple transducers are usually expensive, bulky, and complicated. Single-element transducers utilizing amplitude A-mode measurement can be a simple, lightweight and energy-efficient alternative for obtaining a control signal. Sikdar et al. presented [79] a finger motion prediction approach using a set of mechanically actuated single-element transducers. A set of conducted tests indicated that the approach provides high accuracy (98%) of the classification of individual finger movements for a group of able-bodied subjects. Other studies performed on forearm muscles using multiple A-mode transducers also proved applicability and accuracy of these simplified systems for the task of gesture recognition [80,81,82,83,84]. Compared to standard linear ultrasound probes, A-mode transducers can be arbitrarily placed around the targeted muscle groups.

The method shows a comparable or higher spatial resolution when compared [85,86,87,88] with sEMG and possesses multiple key advantages such as immunity to moisture, sweat, and electrical inference. Although it was not tested in the conducted studies, there are reasons to assume that the method is sensitive to changes of the muscle volume caused by post-amputation muscle atrophy [89] in amputees and during physical activities [90] in healthy users. Apart from that, the techniques employing ultrasound imaging are very sensitive to the motion of the probe with respect to the position of the monitored muscles, since even the slightest changes in the position of the probe can drastically change the cross-section view [78]. Multiple approaches have been used to compensate for the shift of the probe. An approach proposed by Yang et al. [91] consisted of manual adjustment of the probe during the preparation stage at each donning of the probe. This calibration step required an image matching algorithm which was used to compare the current placement’s 2D scan with the initial scan (obtained during the gesture classifier training process). During the calibration process, the user maintained the same initial resting hand gesture while researchers manually adjusted the position of the probe until the scans had a high degree of similarity. Research groups of Castellini et al. [75] and McIntosh et al. [78] applied optical flow evaluation for compensation of the probe movement during multiple tests. The undesired motion of the probe was compensated by evaluating the change of the image when the hand was in resting reference position. This method prevented significant drift errors during the experiment and improved prediction accuracy. However, a robust method to compensate for the probe shifts relative to the forearm bones is yet to be developed. 

Due to a large difference in acoustic properties of skin and air, to obtain ultrasound images, a conductive medium should be used to eliminate the layer of air between the ultrasound probe and skin. A commonly used solution is to use ultrasound gel which is often uncomfortable in handling and dries quickly upon exposure to air. This can be partially resolved using hydrogel pads, which, however, also tend to dry. 

Combination of sonomyography with other myographic methods can lead to increased precision of gesture recognition. For example, surface electromyography (can reflect the degree of muscle tension) and ultrasound imaging (can monitor the change of morphological structure of an active muscle) are both non-invasive techniques. Together, they can provide more comprehensive information about movements. Xia et al. [92] implemented a hybrid system, which incorporated four compact modules, each combining sEMG sensor with A-mode ultrasound transducer. Since a simultaneous firing of multiple ultrasound transducers might cause crosstalk, the researchers applied a modulation to the signals. The combined approach showed supremacy over individual methods [92], allowing for achieving 89% overall accuracy for 20 gestures, which was 5% higher than using only SMG and 21% higher than sEMG.

### 3.5. Force Myography

**Figure 6:**
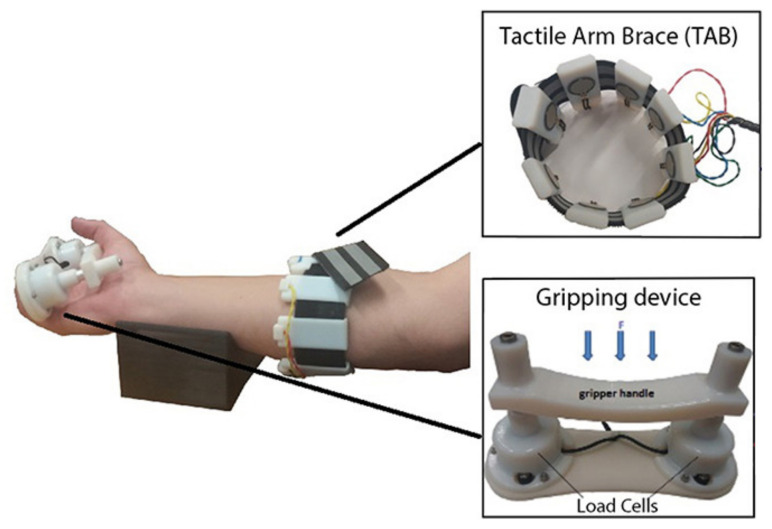
An example of FMG device with an array of eight force sensors (Tactile Arm Brace) placed on the able-bodied user. Adapted from [101], licensed under CC BY 4.0.

The force myography (FMG) approach consists of placing force sensors on the limb surface and utilizing them for detecting the volumetric changes of the superficial muscle groups. It is also possible to detect the volumetric changes caused by the movement of tendons under the skin surface [93,94]. The method can provide information about the user’s intended motion and grip force. Usually, an array of force-sensing resistors is used to achieve better accuracy. However, some closely related implementations use a wide range of special deformation and stretch [95] sensors, such as optical fibre-based sensors [96], capacitance-based deformation sensors [97], Hall-effect based deformation sensors [98], and barometric sensors [99]. Another interesting variation of the approach was introduced by Jiang et al. [100], where the sensing system utilized a thin array of adhesive stretchable deformation sensors (e-skin) on the dorsal side of the hand to recognize a set of 10 gestures with 94% accuracy. Regardless of the sensor type, the array of sensors must be customised effectively to achieve a high-quality capture of muscle deformation while accommodating the wide variation in residual limb geometry, musculature, socket fit, and skin conditions. The array of force sensors is typically placed close to the wrist or closely to the targeted muscle group (finger flexors) as shown in Figure 6. 

There have been numerous studies that investigated the feasibility and accuracy of the approach [102,103,104,105]. Cho et al. demonstrated [106] the feasibility of this control approach for hand prosthesis control. As in the case of EMG, an increased number of sensors may provide better accuracy for the task of gesture recognition. A wearable device incorporating high-density sensor array of 126 force-sensitive resistors located in 14 rows, presented by Radmand et al. [103], demonstrated superior accuracy (comparing to devices with a small number of sensors) allowing for achieving 99% accuracy for a set of eight gestures. Researchers additionally noted that even a small load, when applied to the device, could drastically reduce its accuracy; this remains an unresolved challenge. 

The FMG method has inherent drawbacks, such as poor spatial resolution caused by adjacent muscle crosstalk and susceptibility to muscle fatigue. Jiang et al. [107] proposed a co-located approach for capturing both EMG and FMG simultaneously at the same location. Combining these two sensing methods significantly increased the classification accuracy as compared with either of the single sensing modalities.

### 3.6. Phonomyography

Phonomyography (PMG, mechanomyography, acoustic-myography, vibromyography) is a non-invasive method of detecting muscle activity based on the measurement of low-frequency oscillations caused by morphological changes in muscle fibres during their contraction. During the start of the muscle movement, the low-frequency peaks are caused by large changes in the muscle shape. The subsequent vibrations are caused by the oscillations of the muscle fibres at the muscle’s resonant frequency [108]. These vibrations in the range of 5 to 100 Hz (with displacement amplitude up to 500 nm [109]) can be detected using microphones or low-mass accelerometers placed on the skin over the belly of the muscle. The vibration can also be measured with a non-contact approach, such as laser Doppler vibrometry [110,111,112]. As a control signal for externally powered prostheses, PMG possesses several advantages over myoelectric control, including robustness to changing skin impedance, moisture level, and does not require a reference signal that is common to electromyography [113]. However, PMG signals are susceptible to ambient noise, adjacent muscle crosstalk, and sound artefacts caused by the motion of the sensor [114,115].

One of the first examples of an implementation of a practical PMG-based detection system of muscle contractions for prosthesis control was presented by Silva et al. [114,116]. The system used three microphone-accelerometer sensor pairs to record PMG signals and movement around the distal end of the residual limb of a below-elbow amputee. Another example of the application of PMG for transradial prosthesis control using six micro-electromechanical microphones was presented by Wilson and Vaidyanathan [115] achieving 62% and 93% accuracy for two sets of five gestures. Esposito et al. implemented a PMG measuring system using a combination of a force sensor with a rigid mechanical coupler since a direct application of the sensor on the skin did not provide full transfer of the vibrations to the sensor [117]. The frequency response of the force sensor with a coupler was enough to measure the PMG accurately. Studies conducted by Lei et al. [118] and Ni et al. [119] show that PMG has a close relationship with the force produced by a muscle since vibratory signals become more prominent with increasing force. 

Since the signals measured by EMG and PMG do not interfere with each other, a fusion of these methods represents another hybrid approach which can provide complementary information about muscle activity [120]. Gregori et al. developed a compact composite probe comprised of two EMG sensors and two identical piezoelectric membranes to be positioned on the monitored muscle [121]. The composite probe allowed the simultaneous differential recording of PMG and EMG signals from the same muscular site providing higher overall accuracy. Considering the relatively low accuracy of PMG-based gesture prediction, it is assumed that further development will be directed towards implementation of hybrid approaches, since PMG is not adequate to be independently used for control of dexterous prosthetic devices. 

## 4. Promising Control Approaches

This section covers promising approaches which represent potential solutions for prosthetic control, although they have not been tested for this task yet. They are separated into two groups according to the provided proprioceptive feedback to the user. The first group does not provide proprioceptive feedback and can be implemented non-invasively. Methods enabling proprioceptive feedback utilize mechanoreceptors in the patient’s residual limb and are potentially capable of invoking a natural sense of movement. However, these methods require surgical procedures.

### 4.1. Control Approaches without Proprioceptive Feedback

The methods presented in this subsection are non-invasive, and they do not provide any sensory feedback to the user. 

#### 4.1.1. Capacitance Sensing

Capacitance sensing is similar to the EIT technique for measuring distances of nearby conductive objects by measuring the capacitance between the sensor and the object and uses a pair of electrodes (transmitter and receivers). The difference between EIT and capacitive sensing lies in the type of measured signal, the way of connecting the sensors to the human body, and the way the obtained data is processed. When the transmitter electrode is excited by a sine wave signal at a fixed frequency (hundreds of kilohertz, depends on the expected capacity of the monitored volume), the receiver electrode is used to measure this wave (the closer the conductive object is, the higher is the received wave amplitude). The magnitude of the received signal is proportional to the frequency and voltage of the transmitted signal, as well as the capacitance between the electrodes (which work as capacitor plates), which in turn depends on the position of the conductive objects between electrodes. The function of the electrodes is fixed, one provides excitation, and the others perform the measurement. The method requires a non-conductive connection between the electrodes and the part of the user’s body. Since the capacitance may be measured for opened gaps, the contact of the skin with the electrode surface is not required. However, the assumption is that there is no significant movement of the measured object in the space between the electrodes.

**Figure 7:**
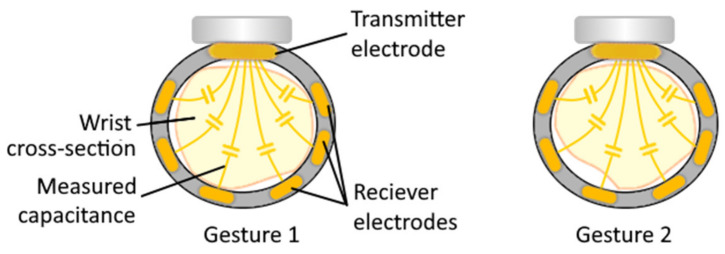
GestureWrist [122]—illustration of the principle. The device measures cross-sectional capacitances between each receiver electrode and transmitter electrode. The cross-section of the user’s wrist changes due to the change of hand gesture.

The primary sensitivity of the gesture recognition devices based on capacitance measurement is to the deformation of the electrodes during movements [97]. However, this approach can also acquire signals related to subtle muscle and surrounding tissue movements deep underneath the electrode array, allowing to monitor complex hand and finger gestures, or intended gestures for amputees (an illustration of the approach is shown on Figure 7). An example of such wearable device is GestureWrist developed by Rekimoto [122] enabling recognition of two gestures (the accuracy was not reported). It was noted that the absolute values measured by the capacitive sensors gradually change over time (leading to a decrease in accuracy) since the position of the wristband was not stable. 

A similar system consisting of an armband with four integrated electrodes developed by Cheng et al. [123,124] showed 58% accuracy for recognizing 36 motion patterns. A limiting factor in those works was a relatively small number of electrodes, which narrowed the number of recognised gestures and limited the overall accuracy. The method’s disadvantages are similar to those of EIT approach: it is susceptible to sweating, electrodes displacement, and parasitic capacitance from other components of the measuring device (there is also the influence of ambient temperature changes). In addition, as in the case of EIT, it can be assumed that an increased number of electrodes might help in increasing the accuracy of gesture prediction. 

#### 4.1.2. Optical Myography

**Figure 8:**
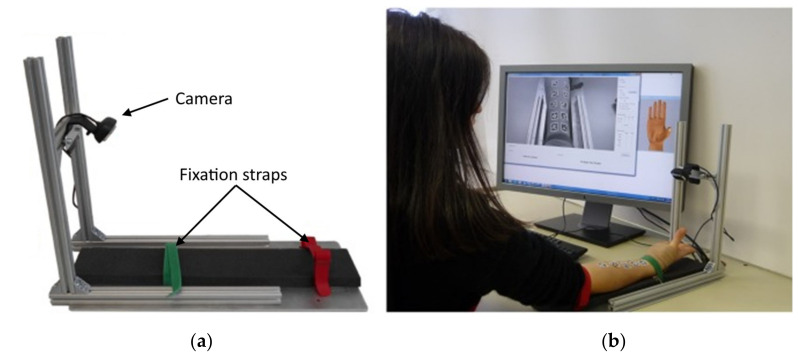
Optical myography experimental setup [126]. (**a**)—rig for stable fixation of the user’s hand. (**b**)—example of data acquisition during the experiment, visual fiducial markers are placed on the ventral side of the user’s forearm. Adapted from [126], licensed under CC BY 4.0.

Nissler et al. studied the potential use of an optical myography (OMG) approach for hand gesture recognition, in which a single low-resolution camera (1280 × 720 pixels) monitors the forearm skin surface deformation caused by the underlying muscle contraction using fiducial marker-based tracking methods [125,126]. The human skin offers very little texture, and therefore it is challenging to register relatively small deformations using visual information. Thus, the implementation task was simplified using a set of 10 adhesive visual fiducial tags uniformly placed in two rows over the ventral side of the monitored forearm. Another significant simplification was used during the conducted tests—the forearm was kept fixed on the table using a harness (see Figure 8). 

Although the conducted studies [127,128] have indicated that the accuracy of OMG is comparable to those of other myographic methods for a small set of detected gestures (achieving up to 98% accuracy for eight gestures [128]), it is apparent that the main drawback is an inability to reliably recognize the changes caused by deep musculature, thus limiting the range of the recognizable gestures [125]. This approach may also be unpractical for use with standard prosthetic sockets since they often use vacuum fitting [129], which will make it impossible to monitor the deformation of the skin surface. Another area of concern is the required precision of localization of the fiducial tags placed on the skin surface. The principle also requires proper illumination of the fiducial tags and minimization of ambient light. Since the most change of the muscle volume manifests as skin movement in the direction normal to the surface, it may be required to place monitoring cameras askew to the skin surface to make measurement possible. This, however, may further complicate the development of a compact solution for integration into a prosthetic socket, which is yet unsolved. It is also questionable whether the accuracy of the approach will be sufficient for a minimum set of gestures when used in a wearable system.

#### 4.1.3. Magnetomyography

Magnetomyography [130] (MMG) is a myography technique based on measuring the magnetic counterpart of the EMG signal—low amplitude magnetic fields (in the scale of pico- and femtotesla) produced by the electrical currents propagating through muscles during contractions. The method is closely related to EMG since according to the Maxwell–Ampere law, a time-changing electrical current generates a magnetic field. However, the ease at which the EMG signal can be recorded and the similarity between characteristics of both signals have encouraged clinical communities and manufacturers to favor the EMG technique. The placement requirements of the sensors are very similar to EMG due to the physical basis of the method. However, the measuring systems such as SQUIDs are cumbersome and cannot be placed on the muscle locations the same way as sEMG electrodes. MMG is less sensitive to the shifts of the sensor than EMG because the magnetic permeability of biologic tissues has a negligible variance (EMG measurements are affected by the high differences in the conductivities of the surrounding tissue) [131]. Another potential advantage of MMG is the possibility of registering the information about the the magnetic field vector generated by the muscle, while sEMG is restricted to the plane of the skin and cannot provide such information [131]. Since MMG sensors do not need electrical contacts with the muscle tissues, they potentially can be covered with biocompatible material making it safer for implantation [132].

Biomagnetic signals are typically very weak and thus are prone to pollution from environmental magnetic noise. Hence, MMG measurements are mostly performed using Superconducting Quantum Interference Devices [133,134] (SQUIDs). However, the high cost of the devices and the complexity of the setup (requiring a temperature-controlled environment with the removal of the environment noise) limit the spread of this sensing technique. Multiple biomagnetic sensing techniques offer more straightforward implementation when compared with SQUIDs, such as highly sensitive Hall-sensors [132,135,136], Optically Pumped Magnetometers [137] (OPM) and other [132]. Heidari et al. [135] implemented a compact CMOS Hall-sensor, potentially making MMG more affordable for use in wearable devices and prosthetics. However, a system for noise-cancellation remains unsolved. Zuo et al. continued developing highly sensitive compact sensors for MMG and discussed a potential implementation of miniaturised MMG systems for further implantation into muscles [132,136] (however, this was not clinically tested).

The MMG approach is still far from practical applications since multiple shortcomings must be dealt with. The main challenge is to isolate the weak biomagnetic signals from the noise of the geomagnetic field. It is to be noted that, in all human studies, the MMG signals were recorded while the participants performed isometric contractions with their muscles [130,138]. Significant morphological changes of the skeletal muscle during muscle contraction may affect the quality of the MMG signal, and this factor requires further investigation.

Currently, MMG does not represent significant diagnostic advantages over multi-channel EMG measurement. On the contrary, the required measurement setup is much more complicated. Further development of this technique is highly dependent on the development of miniaturized, low-cost, and room-temperature magnetometers with a high signal-to-noise ratio and the ability to cancel the geomagnetic field in real-time.

### 4.2. Control Approaches Enabling Proprioceptive Feedback

The methods presented in this section represent concepts of control approaches, which potentially can enable conveying the force applied to the prosthesis back to the muscles enabling natural functioning of the muscle proprioceptors.

#### 4.2.1. Cineplasty

Cineplasty is a surgical technique for direct mechanical linking of muscle in the residual limb to the prosthesis (for example, using a Bowden cable). The approach was first fully described and applied by Vanghetti and later by Sauerbruch at the beginning of the 19th century [139]. While no longer popular, the original approach presented serious disadvantages such as dermatological complications with repeated infections and worsened aesthetic appearance [140]. Additionally, with the typical approach, the muscle excursion and power are diminished since the connection with a Bowden cable was usually connected not at the distal extreme of the muscle. Although representing a number of disadvantages, the method possesses a key advantage that is not available for most of the modern control methods—proprioceptive feedback with the restoration of agonist–antagonist muscle connections. The position, the velocity, and the forces that are applied to the prosthesis are transferred to the muscles, stimulating proprioceptors of the muscle body (and tendons) thus enabling intuitive proprioceptive feedback for the user, which in turn enables more accurate control over the prosthesis.

More modern experimental approaches based on cineplasty imply electromechanical amplification of the force produced by the attached muscle of the residual limb. The basic idea of direct coupling of a residual intact joint motion (or the remnant muscles) to a prosthetic component and enabling natural proprioceptive feedback was described by Simpson in the concept of Extended Physiological Proprioception [141,142] (EPP). The position and movement of a prosthesis joint are at all times directly related to the position and movement of an anatomical joint (or the remnant muscles), which in turn is physically constrained to maintain a constant relationship between it and the prosthesis. An example of implementation of such prosthesis control system was presented by Weir et al. [143]. The prosthesis is controlled by remnant forearm muscles directly attached to a force sensor using exteriorised forearm tendons. The signal obtained by the sensor was used to control a single DOF of an externally powered prosthesis. The patient was able to distinguish between large and small objects with 100% accuracy and between small, medium, and large objects with 80% accuracy. Since the signal could be arbitrarily amplified, this approach eased the control allowing the amputee to use smaller input forces to operate the device.

**Figure 9:**
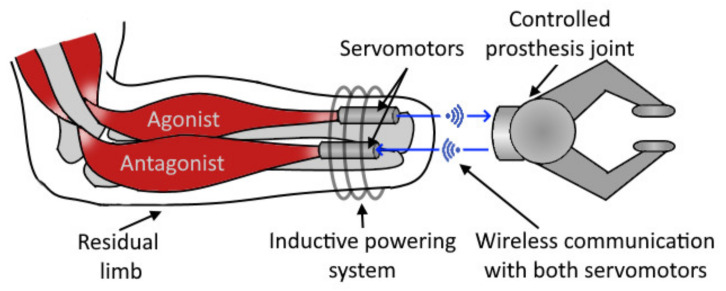
Illustration of the concept of Biomechatronic Extended Physiological Proprioception [147].

A concept presented by groups of Mablekos-Alexiou et al. [144], Kontogiannopoulos et al. [145,146], and Koukoulas et al. [147] further extend the idea of a modern approach to cineplasty. The researchers in a series of papers presented and tested a concept of a novel control topology (Biomechatronic EPP, see Figure 9), proposed to eliminate the aesthetic drawback of cineplasty and Bowden cables. In the proposed approach, the bi-directional force feedback between prosthesis and muscles is enabled using implanted leadscrew driven servomotors with force sensors connected to the remnant agonist-antagonist muscle groups and fixed to the forearm bones. The approach also implies wireless communication and charging between the controlled prosthesis and the implanted feedback control system, thus mitigating possible dermatological complications compared to traditional cineplasty surgeries.

However, the approach is only in the phase of concept, and multiple challenges are yet to be solved, such as a safe connection of the muscles with mechanical components.

#### 4.2.2. Myokinetic Control

**Figure 10:**
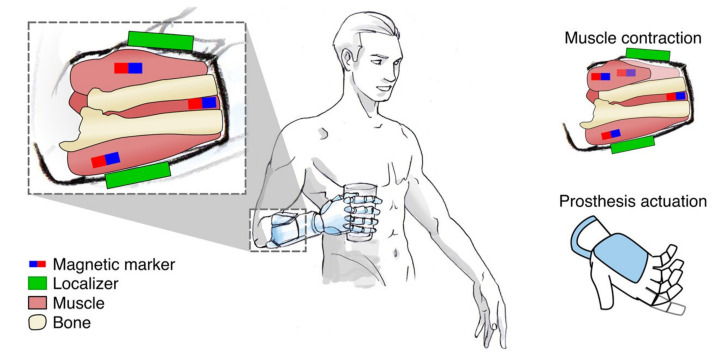
Concept of Myokinetic control interface [148]. Magnetic markers are implanted in remaining wrist muscles; voluntary contractions of these muscles are monitored by three-axis magnetic field sensors which estimate the position of the magnetic markers. This information is used for proportional control over degrees of freedom of a prosthetic hand. Reproduced from [148], licensed under CC BY 4.0.

Myokinetic control interface (further referred to as MYKI) is a concept proposed by Tarantino et al. [148] as part of the MYKI research project [149]. The control method is based on sensing the magnetic field of permanent magnet markers directly implanted into remnant muscles (see Figure 10).

Localizing the position of the magnet is equivalent to measuring the morphological changes of the muscle in which it is implanted: as the magnet moves with the muscle, it gives compound information about the position of the joint and the force applied by the muscle. When the muscle is blocked from movement (for example by an object held by the hand), it performs an isometric contraction, which manifests mainly as radial deformations (changes of muscle’s thickness). During unblocked movements (isotonic contractions), the morphological changes in the muscles are mostly observed in the axial direction (change of muscle’s length). By monitoring both radial and axial deformations of the muscles, it is possible to retrieve information about the force exerted by the muscle and use it to control a prosthesis joint. The magnetic fields generated by the magnetic markers are detected by external three-axis magnetic field sensors placed around the residual limb in the prosthesis socket. The technique is similar to MMG; however, in the case of MYKI, the magnitude of the magnetic field is defined by the chosen material of the markers. For initial testing of the approach, the researchers developed a forearm mock-up with simulated extrinsic muscles of the hand and experimentally assessed the performance of six three-axis sensor localisers tracking positions of four markers implanted in the mock-up muscles.

The goal of the MYKI research project [149] is to develop a bidirectional human–machine interface enabling the users with an intuitive direct control over multi-DOF prostheses. Force and position feedback can be possibly enabled with an actuator that can exert a force on the magnetic markers from outside the body (using external magnetic fields). Since several types of sensory receptors lie within skeletal muscles, the force applied to the muscles can be sensed by these proprioceptors. MYKI, from its definition, is closely related to the cineplasty approach since the bidirectional magnetic linking with the muscles is basically a contact-less implementation of the cineplasty principles. For this reason, the method potentially possesses the main advantage of cineplasty, which is the perception of force and position. Currently, the researchers are investigating the idea to embed electromagnets within the prosthetic socket to generate a controllable external magnetic field, large enough to interact with the implanted permanent magnets [149]. Researchers are also exploring the safety and biocompatibility of the implantable magnetic markers. Even though common magnetic materials are not biocompatible, some manufacturers offer biocompatible coatings for the produced magnets.

In the following works [150,151], researchers assessed the accuracy of the position tracking of multiple magnetic markers with a set of magnetic field sensors. It was found that increasing number of magnets (and decreasing available space between them) causes localization errors and false predictions to occur more often. Other error factors include the geomagnetic field, external magnetic fields produced by local magnetic sources, and, potentially, the effects of relative misalignments between the socket and its initial position. The latter suggests that the problem of socket misalignments will require the development of a calibration procedure or a dynamic compensation similar to what is required for SMG and OMG methods. This approach would benefit from developing a system for compensation of magnetic noise, similar to what is required for MMG.

## 5. Enabling Intuitive Proprioceptive Feedback

The absence of proprioceptive feedback, along with the absence of touch sensations is one of the most critical inconveniences of modern prostheses [13]. In most cases, the user has to coordinate the movements of the prosthesis and adjust the grip using relying on exteroception (usually visual feedback). According to the assessment of user-needs [13], force feedback is considered to be critical as it cannot be estimated visually by the user. Feedback on the position of the prosthetic fingers is also considered essential as it may allow for a decrease in the attention required for handling the prosthesis. From the previously discussed methods of implementing control for hand prostheses, only two concept approaches are potentially capable of utilizing the remaining native proprioceptors in the stump: cineplasty and myokinetic control. The rest of the methods are incapable of that, at least in their initial definition. This section will briefly report on advances in enabling intuitive proprioceptive feedback for possible use with remaining control approaches. It is to be noted that currently there are no methods to fully replace this sensory information (or to decode it from the neural signals) from the amputated limb, and the methods presented here are only utilizing the remaining receptors in the muscles in order to provide users with feedback to at least some extent (for certain DOFs), however requiring the user to adapt to newly provided connections.

Variety of developing techniques enabling brain–machine interface and connection to the neural periphery appear to be the most promising in terms of the long-term development of prosthetics. These approaches [33,152] potentially may provide a way to achieve a completely different level of control and feedback for prosthetic devices, but currently face multiple impediments limiting their use. However, they are related to bidirectional neural interfaces which are not covered in this review and require standalone literature survey. Another group of techniques consists of sensory substitution methods incorporating other modalities (tactile, vibration, skin stretch) for implementation of the position and the force (exerted by of the prosthesis) feedback. However, these techniques can hardly be called intuitive as they involve the transformation and rerouting of sensed information from the prosthesis to an available sensory channel of the user (potentially of a different modality) at a free site of the stump. Provided stimulation in the long-term may be obtrusive and cause discomfort to the user. These factors were pointed-out as undesired in user needs assessment [13] performed by Peerdeman et al. Apparently, this approach requires extensive training since the information is perceived by completely different sites than before amputation. Additionally, these techniques cannot engage remnant muscle proprioceptors, which are important for natural kinaesthetic perception [17]. These techniques are closely related to the topic of non-invasive sensory feedback and are covered by works of Stephens-Fripp [31].

### 5.1. Agonist–Antagonist Myoneural Interface

**Figure 11:**
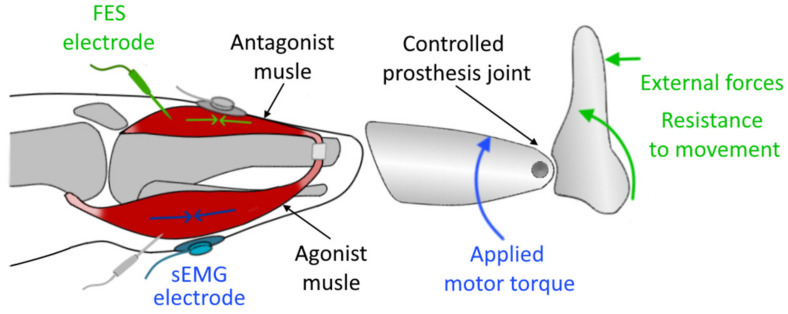
Agonist–antagonist Myoneural Interface (AMI) [153] for leg prosthesis. Volitional contractions of both agonist and antagonist muscles are recorded using an sEMG technique and converted into a control signal for the actuated joint of the prosthesis. The patient can stiffen the joint by simultaneously contracting both muscles of the AMI. The force feedback from the prosthetic joint is provided to the user via artificial contractions of the antagonist muscle, which are triggered by a Functional Electrical Stimulation (FES) generated by the prosthesis controller. These artificially imposed contractions of antagonist muscle create a natural sensation of a load in the volitionally contracted agonist muscle.

Agonist–antagonist Myoneural Interface [153] (AMI, see Figure 11) represents a possible way of providing proprioceptive feedback for users of prosthetic devices. It consists of a control and feedback system utilizing an agonist-antagonist muscle group for both obtaining the control signal for the prosthesis and enabling the feedback.

The concept of AMI was proposed by Clites et al. [154]—primarily developed and tested for lower limb prostheses. With some adaptation, it may be utilized for arm prostheses control since it potentially can be combined with any control method extending its capability to communicate the resistance to the movement of the prosthesis and thus improving the feel of an embodiment for the patients. Researchers surgically constructed two agonist–antagonist muscle pairs within the residual limb of patients with a transtibial amputation. In each pair, the muscles were mechanically linked via a tendon, which passed through a synovial canal, harvested from the amputated ankle joint at the time of amputation. These synovial canals were anchored to the tibia and served as a biologic pulley. Each muscle pair was assigned to the task of controlling corresponding joints of a 2 DOF ankle-foot prosthesis. Volitional contractions of the muscles in the pairs were recorded using sEMG. The amplitudes of these signals from the agonist and antagonist muscles of each AMI were interpreted as desired joint torques (produced in the opposite directions), making it possible for the users to stiffen a prosthetic joint by simultaneously contracting both the agonist and the antagonist muscles within the AMI associated with the corresponding joint. Next, the closed-loop torque control over the prosthesis was enabled by creating artificial contractions in the antagonist muscles, which were triggered by a Functional Electrical Stimulation (FES) with fine-wire electrodes placed within the muscles. Since the muscles in agonist–antagonist pair are attached, contraction in one muscle leads to a stretch in the other. Proportional to torque measured on the prosthesis, an FES was applied to the antagonist muscle within each pair, controlling the force borne on the mechanically coupled muscles, which in turn provided the users with a sensation of the force as feedback. Since AMI preserves the native muscle connection, it makes it possible for proprioceptive signals from mechanoreceptors (muscle spindles) within both muscles to be communicated to the central nervous system [153].

The results showed that enabling proprioceptive feedback significantly improved performance during torque control tasks. Moreover, the patients noted an improved feeling of the embodiment of the prosthesis due to improved control over the DOFs. In further work, the researchers showed that the approach is also applicable for higher levels of amputations, such as transfemoral amputation, by surgically creating agonist–antagonist groups with linked reinnervated muscle grafts (RPNI) and applying the AMI control–feedback topology over them. In addition, it was noted that the approach might also improve performance to some extent during torque-control tasks for patients with traditional amputation (without constructed agonist–antagonist pairs). However, stimulating muscles that are fixed isometrically would result in less effective and less intuitive proprioceptive feedback. Since the stimulation impulses generated by a FES may lead to crosstalk and complicate the processing of data from EMG sensors, there is an opportunity to take advantage of other approaches that are immune to electric noise, such as SMG, FMG, NIRS, and PMG.

### 5.2. Kinaesthetic Illusion

**Figure 12:**
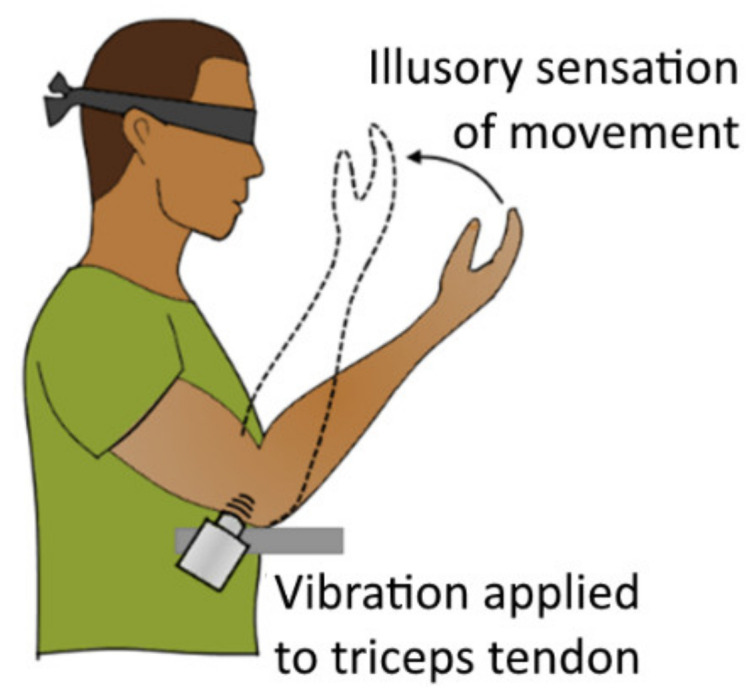
Example of a vibration-induced kinaesthetic illusion of movement: vibration of triceps tendon induces an illusion of elbow flexion. The vibration of muscle tendon activates the muscle proprioceptors and causes an illusion of a sense of joint movement.

Another approach to enabling feedback is the physiological phenomenon of kinaesthetic illusion—a vibration-induced illusory sense of movement of the limb. Goodwin et al. demonstrated [155] that vibro-mechanical stimulation applied externally to muscle tendons induces the human subjects with the illusory perception of limb joint movements by activating muscle spindle endings [156]. The subjects perceive an illusory elongation of the vibrated muscle (thus moving the limb), even though no real movement is actually performed (Figure 12). After the stimulation is stopped, the subject perceives an illusory recovery movement called “after-effect”, which is directed toward the actual position of the limb. In a series of tests, it was shown that vibrations with frequencies in the range of 70–115 Hz with an amplitude in the range around 0.1–0.5 mm are the most effective at creating an illusion of movement [17,157].

Extensive work in this direction was performed by Marasco et al. [17]. In a series of tests, researchers investigated the use of this effect to enable kinaesthetic feedback for patients with upper limb amputation who had undergone targeted muscle and sensory reinnervations for the implementation of a prosthetic control topology. The remaining motor and sensory nerves were surgically redirected to reinnervate other proximal muscles (further utilized for EMG-based control) and skin sites. Throughout the tests, researchers used a vibration of 90 Hz with 500 μm amplitude to stimulate the reinnervated residual muscles of amputee participants to enable kinaesthetic feedback. In the participants’ group, stimulation of muscles reinnervated by the median nerve provided various illusions of finger flexion, while stimulation of the muscles reinnervated by the radial nerve provided illusions of extension. The patients were asked to demonstrate percept illusory movement of the amputated hand by reproducing the movement with the intact hand (which simplified the assessment of the illusory effect). Percept speed of illusory movement was estimated to approximately 52 mm/s when the kinaesthetic illusion was applied actively (simultaneously with a volitional contraction of the corresponding opposing muscle). However, researchers were able to increase the percept speed by showing the participants a visualization of faster arm movement. Researchers also demonstrated the feasibility of having two tactors stimulating in agonist–antagonist muscle pairs, which was used for prosthetic control topology utilizing both these muscles. Another factor investigated during experiments was reflexive muscle contraction as a response for the applied vibrational stimulation, as was described in the work of Eklund and Hagbarth [158]. It was found that the degree of this reflexive contraction is negligible in comparison to a regular (volitional) muscle contraction and does not impact EMG control.

Researchers noted that, in in the future, they plan to investigate the inclusion of additional feedback from multiple limb joints along with the possibility of simultaneous integration of multi-modal sensory feedback.

## 6. Discussion

In this section, we will discuss the presented control techniques for prostheses as well as the challenges and potential directions of future research on the topic.

The described control approaches and concepts represent the latest advances and promising techniques for implementing prosthesis control based on measuring the activity of remaining muscles in the patient’s stump. Despite all the advances in this field, the prosthetic hand rejection rates are estimated to be as high as 45% [52], often because the users are dissatisfied with the lack of sensory feedback and overall intuitiveness of control over the prosthetic device. It is to be noted that, in laboratory conditions, many of the aforementioned methods can achieve high accuracy even for proportional control of individual DOFs of a prosthesis. However, only a small number of techniques would enable accurate and robust control while performing everyday activities.

For clarity, we summarized important aspects of each control method in an overview table (Table 1 and Table 2). The table provides insights on the tested applications of each method, its susceptibility, physical basis, invasiveness, the capability of recording deep muscles, possible sensor placements, and the typical number of channels used for the gesture prediction task. It also represents the combinations of the techniques that were covered in the literature. The overview table does not represent the accuracies of individual methods, since the actual accuracy of gesture prediction based on each of the methods depends on the type of regression or classification models used (such as artificial neural networks, support vector machines, linear discriminant analysis, hidden Markov Models, and others). In general, it can be expected that the number of sensors can drastically influence the accuracy since it can provide better spatial resolution and thus enable extraction of more features [48,103].

Techniques representing a fusion of multiple approaches represent particular interest for future research as they can be a synergy of the strengths of each of the methods and may compensate for their individual weaknesses. Currently, most of the research investigating the possibilities of hybrid sensing techniques concentrates on combining EMG with approaches of different sensing modalities. It can be anticipated that not all the methods covered in this review could be combined for simultaneous measurement. For instance, measurement sequences of EIT and capacitance sensing are anticipated to produce high noise for EMG (both surface and intramuscular) as well as for MMG or even represent a danger for the user in case of combining it with MYKI, due to the excitation signal produced by the sensors. Electric inference excited by an EIT device during EMG measurement may be avoided by utilizing signal filters or performing the measurements in sequences. The possibility of combining SMG with PMG methods depends on the sensitivity of the utilized vibrometers. Combining ideas of SMG and myokinetic control approaches may be envisioned as muscle-implantable markers (with high acoustic impedance), which may greatly simplify image processing and feature extraction for SMG allowing for more clearly distinguishing individual muscle movements. Generally, the feasibility of the hybrid approaches will also depend on the actual size of the sensors, the available space, and residual structures of the residual limb. In order to provide a compact solution, the systems have to be developed as a single device instead of separate units.

Considering that a vast majority of the abovementioned methods (except for cineplasty) rely on the measurement of physiological parameters of muscles at a particular location, they are susceptible to sensor-shifts between multiple sessions of use and thus require the development of a robust calibration procedure or a continuous shift compensation method in order to retain the accuracy during daily movements. EMG, EIT, MMG, PMG, FMG, NIRS, and MYKI approaches require a calibration procedure that takes into account the position of the sensor with respect to the forearm bones, since the skin might not visually provide a robust estimation about these factors due to possible deformation. Methods capable of mapping tissue distribution in a cross-section plane of the forearm (such as EIT, SMG) potentially offer information regarding the position of the ulna and radius, which may be used as a reference for shift compensation. Osseointegration [159,160] is a possible alternative making calibration procedure unnecessary by enabling robust reference for fixation of the sensors; nevertheless, the method currently possesses several issues which are to be solved. Since sensor shifts may also be related and dependent on the orientation of the limb in space, some studies [161,162,163] successfully applied inertia sensors for tracking the relative orientation of the prosthesis by training the muscle activation classifier with additional inputs from these sensors.

Regarding the approaches of enabling the proprioceptive feedback, AMI seems to be the most intuitive since it more closely resembles a natural interaction of agonist–antagonist: muscles stimulated by FES work as if they were mechanically linked to the prosthetic components (similar to the cineplasty approach, where force is transferred by Bowden cables). However, currently, no studies are available comparing the actual impacts of these approaches for prosthesis users. In general, it is often considered that further development in the field of the prosthetics should continue in the direction of developing intuitive feedback systems [13].

## 7. Conclusions

The goal of this review was to examine the approaches for implementing control over hand prosthesis, describe their weaknesses, important aspects and to discuss areas of exploration for the future. Current research and development efforts in invasive, and non-invasive approaches were explored; however, the focus of the review was limited to the approaches based on the measurement of the activity of remaining muscles in the residual limb.

Even though modern prostheses use the latest drive systems and modern materials, it should be borne in mind that the mechanical design aspect of a prosthesis is not the main factor that affects the intuitiveness of its use. A much more essential aspect is a sensory feedback system, which provides the sensation of touch, position, speed of movement and exerted force in the prosthetic limb, with which a person can perform coordinated movements without having to continually monitor the device visually. Only a limited number of control approaches enable intuitive proprioceptive feedback. However, there are techniques, such as agonist–antagonist myoneural interface [27,153] and kinaesthetic illusion [155], that potentially may enable proprioceptive feedback for several aforementioned approaches.

Prosthetics is an ever-evolving field, combining the state-of-the-art knowledge from areas of science such as material science, robotics, neurology, and many others. The development of new, improved prosthetic devices is a matter that significantly affects the lives of people with disabilities. Given the complexity of the human hand as a system, it cannot be expected that the prosthetic limbs will be soon comparable to their biologic analogue. However, researchers have been applying many creative techniques that significantly improve the lives of people using prosthetic devices.

## Figures and Tables

**Table 1:** Comparative overview of the control methods.

Name of the Method	Measured Property	Applications	Provides Proprioceptive Feedback	Requires Surgical Procedure	Requires Contact of Sensor/Electrodes with Tissues
*Electromyography* *(EMG, sEMG, iEMG)*	Muscle electric potentials	Prosthesis control, Hand gesture prediction (up to 27 gestures [48])	No	sEMG–no iEMG–yes	Yes
*Electrical impedance tomography (EIT)*	Tissue impedance	Prosthesis control, Hand gesture prediction (up to 8 gestures [60])	No	No	Yes
*Near-infrared spectroscopy (NIRS)*	Tissue oxygenation (through the amount of scattered light)	Prosthesis control, Hand gesture prediction (up to 9 gestures [66])	No	No	Yes
*Sonomyography* *(SMG)*	Change of muscle morphology	Prosthesis control, Hand gesture prediction (up to 15 gestures [76])	No	No	Yes
*Force myography (FMG)*	Change of muscle morphology measured on the skin surface	Prosthesis control, Hand gesture prediction (up to 8 gestures [103])	No	No	Yes
*Phonomyography (PMG)*	Muscle fibre oscillations	Prosthesis control, Hand gesture prediction (up to 5 gestures [115])	No	No	No (possible contactless measurement [110])
*Capacitance sensing*	Tissue capacitance	Hand gesture prediction (up to 2 gestures [122])	No	No	No
*Optical myography (OMG)*	Change of muscle morphology measured on the skin surface	Hand gesture prediction (up to 8 gestures [128])	No	No	No
*Magnetomyography* *(MMG)*	Magnetic fields generated by muscle	Concept	No	No (possible implantation)	No
*Cineplasty*	Muscle length	Prosthesis control (original approach), Concept	Yes	Yes	Yes
*Myokinetic control* *(MYKI)*	Change of muscle morphology (through magnetic fields)	Concept	Yes	Yes	No

**Table 2:** Comparative overview of the control methods (continued).

Name of the Method	Sensors/Electrodes Placement	Susceptibility	Monitoring Deep Muscles	Tested Hybrid Approaches	Typical Number of Channels/Electrodes
*Electromyography* *(EMG, sEMG, iEMG)*	Over/inside targeted muscle	Sweating, Muscle fatigue, Electromagnetic noise	sEMG–no, iEMG - yes	NIRS, PMG, FMG, SMG	2–32, up to 192
*Electrical impedance tomography (EIT)*	Over targeted muscle, Over related tendons	Sweating, Electromagnetic noise	Yes	–	8, up to 64
*Near-infrared spectroscopy (NIRS)*	Over targeted muscle	Ambient light, Muscle fatigue	No	EMG	2–4
*Sonomyography* *(SMG)*	Over targeted muscle, Over related tendons	Probe shift	Yes	EMG	Probe with 128 transducers (up to 512) A-mode: 4 transducers
*Force myography (FMG)*	Over targeted muscle, Over related tendons	Muscle fatigue	No	EMG	8, up to 126
*Phonomyography (PMG)*	Over targeted muscle	Ambient acoustic noise, Adjacent muscle crosstalk, Sensor movement	No	EMG	6
*Capacitance sensing*	Over targeted muscle, Over related tendons	Sweating, Electromagnetic noise	Yes	–	3 (receiver electrodes)
*Optical myography (OMG)*	Over targeted muscle, Over related tendons	Ambient light, Muscle fatigue	No	–	Single camera, 10–18 skin markers
*Magnetomyography* *(MMG)*	Over/inside targeted muscle	Magnetic interference	No	–	–
*Cineplasty*	Wrist	Infections	Yes	–	Single sensor for each muscle pair
*Myokinetic control* *(MYKI)*	Over targeted muscle	Magnetic interference	Yes	–	6
